# Late Administration of Remdesivir, Guided by Antigen Test Results, in a Patient Experiencing Difficulty Weaning From the Ventilator Due to Severe COVID-19 Pneumonia

**DOI:** 10.7759/cureus.89181

**Published:** 2025-07-31

**Authors:** Keisuke Mataichi, Takuo Yoshida

**Affiliations:** 1 Division of Intensive Care, Department of Emergency and Disaster Medicine, Kashiwa Hospital, The Jikei University School of Medicine, Tokyo, JPN

**Keywords:** antigen testing, antiviral therapy, mechanical ventilation, remdesivir, respiratory failure, severe covid-19 pneumonia

## Abstract

A 70-year-old woman with a history of malignant lymphoma (in remission) and systemic sclerosis required mechanical ventilation because of severe coronavirus disease 2019 pneumonia. Despite the administration of broad-spectrum antibiotics, antifungal agents, and corticosteroids, respiratory failure persisted, and a tracheostomy was performed on hospital day 17. Ventilator weaning remained difficult up to hospital day 30 and persistent detection of the severe acute respiratory syndrome coronavirus 2 antigen prompted initiation of remdesivir. Treatment was continued until the antigen became undetectable, after which successful ventilator weaning was achieved. This case suggests an association between prolonged antigen presence and sustained respiratory failure.

## Introduction

Coronavirus disease 2019 (COVID-19) exhibits cyclical patterns of outbreak and remission, prompting the investigation of various therapeutic approaches [1]. However, current evidence regarding pharmacological interventions for patients with severe COVID-19 pneumonia remains limited [2], and the underlying pathophysiological mechanisms are not yet fully understood. In severe COVID-19 pneumonia cases, an excessive immune response following infection may contribute to pulmonary damage [3]. Accordingly, corticosteroids are administered to counteract the exaggerated inflammatory response, and an interventional study reported that dexamethasone may contribute to a reduction in mortality [4]. However, the clinical relevance of antiviral therapies targeting direct viral mechanisms in severe COVID-19 pneumonia remains unclear [5]. The Society of Critical Care Medicine in the United States of America has issued a recommendation against the use of remdesivir for treating severe COVID-19 pneumonia with mechanical ventilation [2]. Furthermore, because remdesivir was administered at an early stage following a confirmed diagnosis of COVID-19 in clinical trials [5,6], its efficacy when given during the later stages of the disease remains uncertain.

Herein, we report a patient with severe COVID-19 pneumonia and difficulty in weaning off mechanical ventilation, in which the administration of the antiviral agent remdesivir, guided by severe acute respiratory syndrome coronavirus 2 (SARS-CoV-2) antigen testing, resulted in successful ventilator withdrawal.

## Case presentation

A 70-year-old woman with a history of malignant lymphoma, which has been in remission for the past 10 years; systemic sclerosis; cerebral infarction; diabetes mellitus; and hypertension presented to our hospital with fever and impaired mobility. The patient’s medications included prednisolone, 10 mg/day; sulfamethoxazole-trimethoprim, 400 mg/80 mg/day; alogliptin, 25 mg/day; metformin, 500 mg/day; clopidogrel, 75 mg/day; and alendronate sodium, 35 mg/day. Thirteen days before admission, one household member tested positive for COVID-19.

On the day of admission, the patient developed fever and worsening mobility and was transported to the hospital by emergency services. On arrival, her Glasgow Coma Scale score was E3V4M6, body temperature was 38.0°C, blood pressure was 81/60 mmHg, heart rate was 140 beats/minute, respiratory rate was 34 breaths/minute, and oxygen saturation was 87% while receiving 10 L/minute of oxygen via a reservoir mask. Bilateral coarse crackles were auscultated throughout the lung fields, without asymmetry in the breath sounds. Laboratory tests revealed a marked inflammatory response, with a C-reactive protein level of 17.55 mg/dL (reference range: <0.14 mg/dL) (Table 1) and a white blood cell (WBC) count of 157,000/μL (reference range: 3,300-8,600/μL) (Table 2).

**Table 1:** Laboratory findings on admission (blood chemistry)

Parameter, unit	Results	Reference range
Aspartate aminotransferase, U/L	696	13-30
Alanine aminotransferase, U/L	373	7-23
Lactate dehydrogenase, U/L	2,392	124-222
Total bilirubin, mg/dL	0.9	0.4-1.5
Alkaline phosphatase, U/L	98	38-113
Gamma-glutamyl transpeptidase, U/L	31	9-32
Total protein, g/dL	6.4	6.6-8.1
Creatine kinase, U/L	256	41-153
Urea nitrogen, mg/dL	16	8-20
Creatinine, mg/dL	0.88	0.46-0.79
Sodium, mmol/L	133	138-145
Potassium, mmol/L	3.4	3.6-4.8
Chloride, mmol/L	100	101-108
(1→3)-β-D-glucan, pg/mL	6	<10.9
B-type natriuretic peptide, pg/mL	22.9	<18.4
C-reactive protein, mg/dL	17.55	<0.14

**Table 2:** Laboratory findings on admission (complete blood count and coagulation system)

Parameter, unit	Results	Reference range
White blood cell count, /μL	157,000	3,300-8,600
Hemoglobin, g/dL	12	11.6-14.8
Platelet count, /μL	215,000	158,000-348,000
Prothrombin activity, %	85	>70
Prothrombin time-international normalized ratio	1.09	-
Activated partial thromboplastin time, seconds	27.9	24-39
D-dimer, μg/mL	13.1	<1

**Figure 1:**
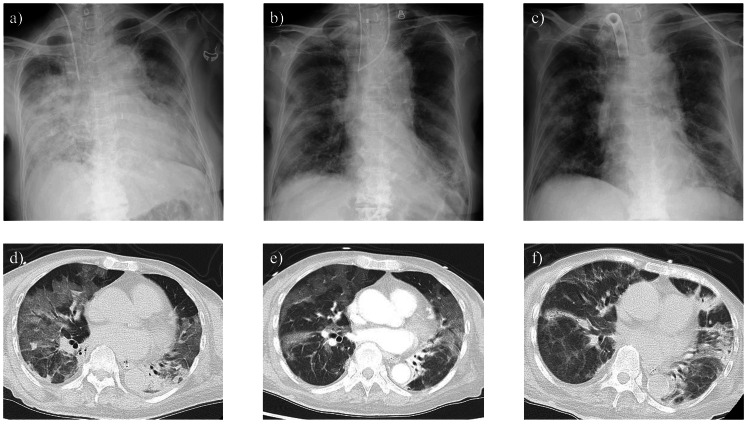
Temporal changes in chest X-rays and chest CT findings. (a,d) Chest X-ray and noncontrast chest CT on day 1, respectively. (b,e) Chest X-ray and contrast-enhanced chest CT on day 11, respectively. (c,f) Chest X-ray and noncontrast chest CT on day 45, respectively CT: computed tomography

A nasopharyngeal specimen tested positive for SARS-CoV-2 by polymerase chain reaction, and the SARS-CoV-2 antigen level was 387 cutoff index (COI) (reference range: <1). Chest X-ray and computed tomography (CT) revealed diffuse ground-glass opacities in both lungs (Figure 1), leading to a diagnosis of COVID-19 pneumonia. On the same day, owing to hypoxemia with 10 L/minute of oxygen via a reservoir mask, as revealed by arterial blood gas analysis (Table 3), tracheal intubation was performed, and the patient was admitted to the intensive care unit (ICU) for mechanical ventilation.

**Table 3:** Arterial blood gas with 10 L/minute of oxygen via a reservoir mask on admission PaCO_2_: partial pressure of carbon dioxide; PaO_2_: partial pressure of oxygen; HCO_3_^-^: bicarbonate

Parameter, unit	Results	Reference range
pH	7.429	7.35-7.45
PaCO_2_, mmHg	29.5	35-45
PaO_2_, mmHg	63.8	80-100
HCO_3_^-^, mmol/L	19.2	22-26
Lactate, mmol/L	5.4	0.5-1.5

**Figure 2:**
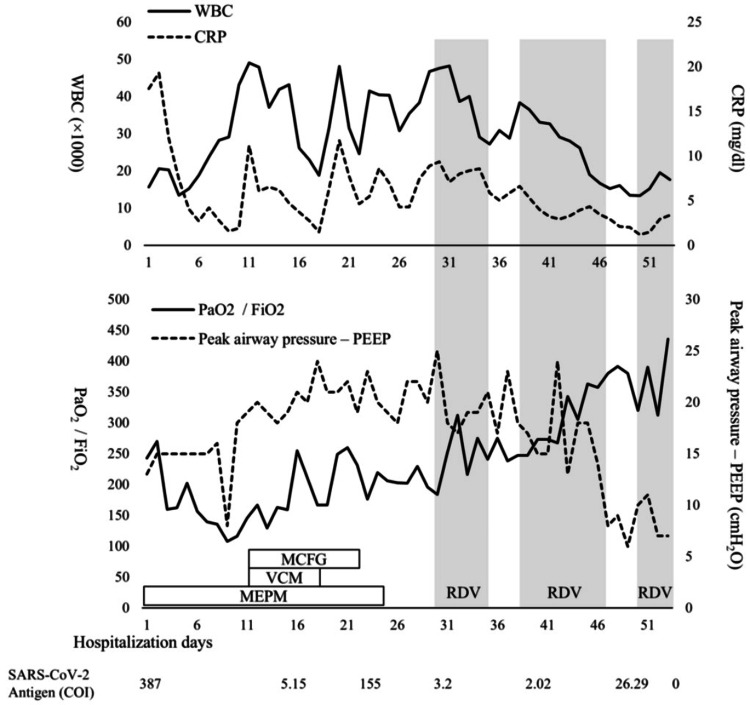
The clinical course of the patient after ICU admission WBC: white blood cell; CRP: C-reactive protein; PEEP: positive end-expiratory pressure; MEPM: meropenem; VCM: vancomycin; MCFG: micafungin; RDV: remdesivir; COI: cutoff index; PaO_2_: partial pressure of oxygen in arterial blood; FiO_2_: fraction of inspired oxygen; ICU: intensive care unit; SARS-CoV-2: severe acute respiratory syndrome coronavirus 2

On the same day, owing to hypoxemia, tracheal intubation was performed, and the patient was admitted to the ICU for mechanical ventilation. The clinical course of the patient after ICU admission is summarized in Figure 2.

The detailed chronological clinical data are presented in Table 4, and the temporal progression of imaging findings, including chest CT and chest X-rays, is illustrated in Figure 1.

**Table 4:** Detailed chronological clinical data a: infection of a cohabitant with coronavirus disease 2019; b: admission to the ICU after endotracheal intubation in the emergency room; c: liberation from mechanical ventilation; d: discharge from ICU; e: discontinuation of remdesivir The ~ in the column head was intended to abbreviate the period to reduce the overall table size COI: cutoff index; SpO_2_: peripheral capillary oxygen saturation; SARS-CoV-2: severe acute respiratory syndrome coronavirus 2; ICU: intensive care unit

Hospital day	-13	~	1	~	10	11	12	13	~	16	~	18	19	~	22	~	24	25	~	29	30	31	~	34	35	36	~	38	39	40	~	48	49	50	51	~	53	54	~	56	~	59
Clinical events	a	-	b	-	-	-	-	-	-	-	-	-	-	-	-	-	-	-	-	-	-	-	-	-	-	-	-	-	-	-	-	-	-	-	-	-	-	c	-	d	-	e
SpO_2_, %	-	-	99	-	95	97	99	96	-	98	-	94	95	-	97	-	99	99	-	95	95	94	-	96	95	97	-	96	99	95	-	98	96	98	99	-	96	99	-	97	-	97
Respiratory rate, /minute	-	-	34	-	20	27	19	24	-	23	-	18	29	-	21	-	22	19	-	20	20	18	-	24	21	24	-	22	22	22	-	34	32	31	27	-	26	22	-	27	-	20
Positive end-expiratory pressure, mmHg	-	-	10	-	7	7	7	8	-	8	-	8	9	-	10	-	10	10	-	8	8	8	-	8	8	8	-	8	8	8	-	5	5	5	5	-	5	5	-	-	-	-
Fraction of inspired oxygen, %	-	-	50	-	60	60	60	60	-	60	-	45	60	-	40	-	40	40	-	40	40	40	-	40	40	40	-	40	35	35	-	35	35	35	30	-	30	30	-	-	-	-
Tidal volume, mL	-	-	365	-	418	422	414	415	-	413	-	408	383	-	324	-	344	360	-	362	399	372	-	411	348	386	-	406	392	363	-	322	373	394	326	-	358	399	-	-	-	-
C-reactive protein, mg/dL	-	-	17.55	-	1.92	11.18	6.09	6.54	-	3.7	-	1.48	6.39	-	4.63	-	6.96	4.29	-	8.88	9.35	7.09	-	8.56	5.85	5.04	-	6.61	5.29	4.03	-	2.08	2	1.18	1.47	-	3.39	2.72	-	2.06	-	1.09
White blood cell count, /μL	-	-	15,700	-	43,200	49,000	47,900	37,100	-	26,100	-	18,800	31,700	-	24,600	-	40,300	30,800	-	46,700	47,600	48,200	-	29,100	27,200	30,900	-	38,300	36,400	33,100	-	16,100	13,400	13,300	15,200	-	17,600	18,700	-	23,000	-	14,300
SARS-CoV-2 antigen, COI	-	-	387	-	-	-	-	1.66	-	5.15	-	-	-	-	155	-	-	-	-	3.2	-	-	-	1.1	-	-	-	2.02	-	-	-	-	-	26.29	-	-	0	-	-	-	-	-
Remdesivir, mg/day	-	-	-	-	-	-	-	-	-	-	-	-	-	-	-	-	-	-	-	-	200	100	100	100	100	-	-	-	200	100	100	100	-	200	100	100	100	100	100	100	100	100
Dexamethasone, mg/day	-	-	6.6	6.6	6.6	-	-	-	-	-	-	-	-	-	-	-	-	-	-	-	-	-	-	-	-	-	-	-	-	-	-	-	-	-	-	-	-	-	-	-	-	-
Meropenem, g/day	-	-	3	3	3	3	3	3	3	3	3	3	3	3	3	3	3	-	-	-	-	-	-	-	-	-	-	-	-	-	-	-	-	-	-	-	-	-	-	-	-	-
Vancomycin, g/day	-	-	-	-	-	1.25	1	1.5	1.5	1.5	1.5	1.5	-	-	-	-	-	-	-	-	-	-	-	-	-	-	-	-	-	-	-	-	-	-	-	-	-	-	-	-	-	-
Micafungin, mg/day	-	-	-	-	-	50	50	50	50	50	50	50	50	-	-	-	-	-	-	-	-	-	-	-	-	-	-	-	-	-	-	-	-	-	-	-	-	-	-	-	-	-

Dexamethasone 6.6 mg/day was administered for COVID-19, and meropenem (3 g/day) was initiated for potential bacterial coinfection. Owing to limited improvement in the respiratory status, vancomycin (1 g/day) and micafungin (100 mg/day) were subsequently added on hospital day 11. Sputum cultures obtained on hospital days 1, 8, and 19 yielded normal flora, and cultures of blood samples collected on hospital days 1, 8, 10, and 19 yielded negative results. Therefore, antimicrobial and antifungal therapies were discontinued on hospital day 24. To investigate alternative causes of respiratory failure, daily portable chest radiography and bedside transthoracic echocardiography were performed. The presence of comorbid conditions, such as congestive heart failure and pneumothorax, was considered unlikely.

The patient's respiratory status remained unchanged after two weeks in the ICU. The ratio of partial pressure of oxygen in arterial blood to the fraction of inspired oxygen persistently remained between 150 and 250, indicating sustained hypoxemia. For adequate ventilation, in addition to positive end-expiratory pressure, an airway pressure >20 cmH_2_O was required to maintain a tidal volume of 6 mL/kg, making ventilator weaning infeasible. Considering this clinical course, tracheostomy was performed on hospital day 17.

To inform decisions regarding infection isolation, SARS-CoV-2 antigen tests were conducted on nasopharyngeal samples collected on hospital days 16 and 29. The antigen levels were 5.1 COI and 3.2 COI, respectively, both of which indicated positivity. All subsequent SARS-CoV-2 antigen tests were performed on nasopharyngeal specimens.

Although remdesivir is not routinely administered for severe COVID-19 pneumonia at our facility, persistent SARS-CoV-2 antigen positivity, sustained elevations of WBC and C-reactive protein levels, and continued respiratory compromise prompted the initiation of remdesivir on hospital day 30 (200 mg/day on the first day, followed by 100 mg/day thereafter). Several attempts were made to discontinue remdesivir upon signs of respiratory improvement; however, each attempt was followed by antigen-level elevation and subsequent respiratory deterioration.

These findings suggested that persistent COVID-19 contributed to respiratory failure. To achieve antigen negativity, remdesivir was reinitiated on hospital day 50, and antigen negativity was confirmed on hospital day 53. Simultaneously, oxygenation and ventilatory status improved, allowing successful ventilator weaning on hospital day 54 and discharge from the ICU on hospital day 56. Remdesivir was discontinued on hospital day 59. The diffuse ground-glass opacities observed in the lung fields on chest X-rays and chest CT scans showed an improvement over time (Figure 1). Complications necessitating readmission to the ICU did not occur thereafter, and the patient was transferred to a rehabilitation facility on hospital day 211.

## Discussion

Herein, we report the case of a patient with severe COVID-19 pneumonia who required prolonged mechanical ventilation. Fluctuations in SARS-CoV-2 antigen levels measured during ventilatory management appeared to reflect the patient's respiratory status; therefore, remdesivir was administered based on the results of SARS-CoV-2 antigen tests. A decrease in antigen levels was accompanied by respiratory improvement, and remdesivir administration was continued until antigen negativity was confirmed, ultimately leading to successful weaning from the ventilator.

The relationship between SARS-CoV-2 antigen levels and disease severity has not been sufficiently elucidated. The COVID-19 virus consists of four structural proteins: spike (S), envelope (E), membrane (M), and nucleocapsid (N), as well as RNA, with the N protein being the most abundantly expressed [7-9]. The SARS-CoV-2 antigen test used at our institution targets the N protein. Previous studies have suggested a correlation between blood antigen levels and disease severity. A prospective observational study by Yalçın et al. reported that circulating N antigen levels were associated with inflammation, organ damage, and ICU admission [10]. Additionally, serum S-antigen levels have been reported to correlate with the need for mechanical ventilation [11]. In the present case, N antigen levels were measured using nasopharyngeal samples, and the observed association between antigen fluctuations and the clinical course was consistent with prior findings. These results suggest a potential association between the SARS-CoV-2 viral load and the severity of respiratory failure.

Evidence supporting antiviral therapy strategies based on the SARS-CoV-2 viral load remains limited. Guisado-Vasco et al. described the use of plitidepsin guided by reverse transcriptase polymerase chain reaction cycle-threshold values, in a patient with chronic myeloid leukemia who developed COVID-19 [12]. On day 48, worsening pneumonia due to persistent infection was observed, prompting the initiation of plitidepsin. Following treatment, improvements in cycle-threshold values and a reduction in oxygen requirements were observed, and therapy was continued with the goal of achieving SARS-CoV-2 negativity. After multiple negative test results were confirmed, the patient was discharged on day 82. Although the type of antiviral agent used and disease severity varied from the present case, both cases involved persistent viral positivity in immunocompromised individuals. Viral persistence may continue even in the late phase, depending on a patient’s immunocompromised condition, suggesting the possibility of sustained direct lung injury.

The clinical course of severe COVID-19 pneumonia is influenced by multiple factors, including underlying comorbidities, immune status, and secondary bacterial infections. A related observational study included a subgroup analysis of ICU cases comparing those who received remdesivir with those who did not. In the nontreatment group, remdesivir was withheld either due to extreme disease severity or delayed presentation after symptom onset [13]. This group demonstrated poorer outcomes in 12-month survival based on multivariate-adjusted survival analysis compared to the treatment group. In the present case report, although remdesivir was administered during a later stage of the illness, respiratory failure improved alongside a reduction in antigen levels. Taken together, these results suggest that, depending on viral load, remdesivir may warrant consideration even in cases with delayed presentation or severe disease. This case highlights a potential clinical approach in which late-phase antiviral intervention, guided by persistent antigen positivity, may still contribute to recovery in critically ill patients when standard early-phase therapies are no longer applicable. However, currently, no antiviral agents have demonstrated definitive efficacy against severe COVID-19 pneumonia in large-scale clinical trials. Future research should assess treatment strategies that consider individual patient immune status and viral load.

## Conclusions

We described the case of a patient with severe COVID-19 pneumonia who initially required prolonged mechanical ventilation and was ultimately weaned following SARS-CoV-2 antigen-level-guided remdesivir administration. This case demonstrated that, even during the late phase of severe COVID-19 pneumonia, remdesivir administration guided by persistent SARS-CoV-2 antigen positivity can lead to clinical improvement and successful ventilator weaning in an immunocompromised patient. These findings underscore the need for further research incorporating patient-specific factors such as immune status and viral load when considering antiviral therapy timing.
